# Lack of evidence for arthropod or vertical transmission of *Hepacivirus bovis* in a persistently infected dairy herd

**DOI:** 10.1007/s00705-026-06747-6

**Published:** 2026-09-28

**Authors:** Nadine Hake, Klaas Dietze, Christian von Holtum, Bernd Hoffmann, Ard M. Nijhof

**Affiliations:** 1 Staatliche Führungsakademie für Ernährung, Landwirtschaft und Forsten, Porschestraße 5a, 84030 Landshut, Germany; 2 Friedrich-Loeffler-Institut, Südufer 10, 17943 Greifswald-, Insel Riems, Germany; 3 Tierärztliche Gemeinschaftspraxis Zeven–Selsingen, Bremer Straße 40, 27404 Zeven, Germany; 4 Institute for Parasitology and Tropical Veterinary Medicine, Freie Universität Berlin, Robert-von- Ostertag-Str. 7, 14163 Berlin, Germany; 5 Veterinary Center for Resistance Research, Freie Universität Berlin, Robert-von-Ostertag-Str. 8, 14163 Berlin, Germany

## Abstract

*Hepacivirus bovis* (BovHepV) has been detected globally since its initial description in 2015, but its transmission routes remain largely unknown. Arthropod-borne or vertical transmission is a common route for several livestock viruses, including Rift Valley fever virus and bluetongue virus. Moreover, many members of the genus *Orthoflavivirus* (family *Flaviviridae*) are transmitted by hematophagous arthropods, raising the possibility that hepaciviruses such as BovHepV, which also belongs to the *Flaviviridae* family, could share similar transmission routes. In this study, we examined possible transmission routes of BovHepV, including the involvement of mosquitoes, hematophagous flies, biting midges, and mites. Arthropods were collected on a dairy farm with documented circulation of BovHepV genotypes 1 and 2 and were subsequently tested for the presence of BovHepV RNA by quantitative real-time PCR. Furthermore, multiple bovine sample types were examined for the presence of BovHepV RNA. To assess local spread, serum samples from cattle on a neighboring farm within flight range of stable flies and mosquitoes were analyzed. Potential vertical transmission was evaluated by testing serum samples from 20 calves and 20 young heifers. None of the analyzed samples tested positive for BovHepV RNA. However, BovHepV was detected in blood samples collected for bovine herpesvirus 1 (BoHV-1) surveillance one month after the arthropod collection periods in both 2022 and 2023, confirming ongoing virus circulation on the farm. Under the conditions investigated, no evidence was found to support transmission via arthropods or vertical transmission. However, rare transmission events or low-prevalence vector involvement cannot be excluded and warrant further investigation.

**Supplementary Information:**

The online version contains supplementary material available at https://doi.org/10.1007/s00705-026-06747-6.

## Introduction

Since the first discovery of a nonprimate hepacivirus in dogs in 2011 [35], the virus has been reported in six continents [4, 8, 12, 15, 19, 40, 55, 68], with a host spectrum ranging from various mammals [7, 23, 36, 50, 66] to ducks [13], sharks [60], and snakes [17]. Hepacivirus is closely related to hepatitis C virus (HCV) and belongs to the *Flaviviridae* family, which also includes the *Orthoflavivirus*, *Pestivirus* and *Pegivirus* genera [49]. The genus currently comprises 14 species that infect a variety of host organisms [https://ictv.global/taxonomy, accessed on 12 January 2026]. This includes *Hepacivirus bovis* (BovHepV), previously referred to as *Hepacivirus N*, that infects cattle and was shown to have a widespread distribution following its initial discovery in 2015 in both Ghana [15] and Germany [4]. The virus has been classified into two genotypes, of which genotype 1 has been further divided into eleven subtypes (A-K). Whereas it is mainly found in cattle, it has also been detected in wild boar, raising questions about its host range and possible zoonotic risk [2, 5, 17, 41]. Infected animals can become persistently viremic, but infections have not been associated with clinical symptoms or elevated liver enzyme levels [5]. Although BovHepV is hepatotropic, with high viral loads found in the liver and associated lymph nodes, and the detection of viral RNA by fluorescent in-situ hybridization in hepatocytes, a clear link between infection and acute liver disease has not been established [4, 5, 26].

The transmission routes for BovHepV remain unknown, although possible routes, including tick-borne transmission, sexual or vertical transmission and iatrogenic transmission by the reuse of needles during vaccination have been proposed [26, 69]. Experimental studies with the related *Hepacivirus equi* (EqHV) demonstrated successful infection of naïve horses by infusion of EqHV-positive plasma [48, 51]. Other members of the *Flaviviridae* family, such as *Pegivirus* and *Pestivirus*, can be transmitted via placental transfer or direct contact with infectious material [39, 45, 56, 61], but BovHepV was not detected in secretions or excretions other than blood from BovHepV-positive cows, suggesting, despite the limited sample size, that alternative transmission routes may be involved [31]. Several members of the *Orthoflavivirus* genus, which also belong to the *Flavivirdae* family, are transmitted by arthropods, including mosquitoes, e.g. West Nile virus (*Orthoflavivirus nilense*) and yellow fever virus (*Orthoflavivirus flavi*), and ticks, e.g. tick-borne encephalitis (*Orthoflavivirus encephalitidis*) and Kyasanur forest disease virus (*Orthoflavivirus kyasanurense*) [6], but arthropod transmission studies for BovHepV have not been reported to date.

In 2020, a dairy farm in Lower Saxony, Germany reported cases of severe respiratory illness in cattle, including pneumonia, bronchitis and/or rhinitis, as well as fever of unknown origin, decreased milk production and peracute deaths. Next Generation Sequencing (NGS) analysis resulted in the detection of BovHepV genotype 1 (subtypes 1 A and F) and genotype 2 in bovine blood samples collected as part of the annual bovine herpesvirus 1 (BoHV-1) surveillance program, but no association with the observed clinical symptoms could eventually be established. No other viral sequences were detected in these samples. Subsequent annual screening for BovHepV revealed an ongoing circulation of BovHepV on the farm, with new infections occurring in animals that previously tested negative, raising questions about the possible transmission route(s) within the herd [31].

To investigate potential modes of BovHepV transmission, arthropods from the affected farm and various biological samples from infected cattle were analyzed. Diagnostic samples of calves developing clinical symptoms during the study time were additionally screened for BovHepV to assess the possibility of vertical transmission. All collected samples were subsequently tested for the presence of *H. bovis* by quantitative real-time PCR (qRT-PCR). To our knowledge, this is the first study investigating potential routes of transmission for BovHepV.

## Materials and methods

### Farm structure and *Hepacivirus bovis* circulation history

The farm maintained approximately 500 cattle distributed across two sites (A and B), located about 2.5 km apart. At Site A, located next to the owner’s residence, approximately 250 lactating and dry cows, predominantly of the Holstein Friesian and Simmental breeds, were kept. After birth, calves were housed individually before being transferred either to a straw-bedded pen with an automatic calf feeder or to group igloos. Female calves aged six to nine months were routinely transferred to Site B, where they were serviced by a bull from the farm’s own herd at 15 to 18 months of age. Animals at Site B were repeatedly examined as part of the BoHV-1 monitoring program. Gestating heifers were subsequently transported back to Site A before calving and integrated into the existing herd after parturition. Heifers kept at Site B consistently tested negative for BovHepV. However, 67 of 162 previously negative animals tested positive for BovHepV in subsequent years following their return to Site A, suggesting the existence of an effective transmission route within the housing system at Site A. As previously reported, genetically identical viruses could be found in different animals, while identical virus strains could persist within the same host for months to years. In addition, reinfections with novel BovHepV variants were observed, including viruses belonging to different subtypes or representing genetically distinct strains within the same subtype [31].

### Collection of clinical samples

Following the identification of *H. bovis* in the herd in October 2020, additional samples were collected in December 2020 from seven cows with the highest virus loads to enable further veterinary diagnostic analyses and to investigate possible transmission routes. This was prompted by the observation that infection was initially confined to animals kept at Site A of the farm, while several previously negative heifers from Site B tested positive after being transferred to Site A. Collected materials included nasal, ocular and buccal swabs, rectally collected faecal samples, pooled quarter milk samples, serum and EDTA blood samples. As the young stock presented with clinical signs including nasal discharge and fever, additional sampling was conducted in February 2021 to exclude BovHepV infection as a potential cause. Serum samples, and occasionally nasal swabs, were collected from 20 calves aged up to five months and 20 heifers aged between five and 24 months.

Furthermore, in 2023, a neighboring bull fattening farm located approx. 550 m from the affected dairy farm was also screened for BovHepV infections. With the farmer’s consent, blood samples collected as part of the annual BoHV-1 surveillance were forwarded by the Lower Saxony State Office for Consumer Protection and Food Safety (LAVES) Oldenburg to the Friedrich-Loeffler-Institute (FLI) for BovHepV analysis.

### Collection of ectoparasites using UV light traps

From May to September 2022, three BG-Pro UV light traps (Biogents, Regensburg, Germany) were positioned both in- and outside the cattle stable near the animals. All traps were installed 1 m above ground level for one week at the end of each month, from 7 pm to 7 am. Traps were emptied each morning. Captured arthropods were cooled in a refrigerator to induce immobility, facilitating the subsequent transfer to 80% ethanol with minimal damage to the specimens. The insects were subsequently identified using standard taxonomic keys [21, 58]. The feeding status of haematophagous arthropods was determined by visual inspection of abdominal blood content using light microscopy and classified as blood-fed, unfed, indeterminate, or damaged. Pools of 25 biting midges from each group were put into separate Eppendorf tubes. For *Stomoxys* flies, the heads and abdomens of half of the specimens were separated and pooled into groups of five per tube. Mosquitoes and black flies (*Simulium* spp.) were individually stored in Eppendorf tubes. All arthropod specimens and tissues were preserved in 80% ethanol. Half of the samples were sent to the FLI for RNA extraction and BovHepV analysis. The remaining half were retained as backup samples at the Freie Universität Berlin (FUB). To verify whether BovHepV was still circulating in the herd at the time of arthropod sampling, blood samples that were collected as part of the mandatory annual BoHV-1 screening were forwarded by the LAVES in Oldenburg to the FLI in October 2023, for *H. bovis* testing using qRT-PCR with the farmer’s permission.

### Collection of ectoparasites using aerial sweep nets

Flies were collected with an aerial sweep net from Monday to Friday during the last week of each month between May and September 2022, between 12 pm and 1 pm. Captured specimens were immobilized by cooling, transferred to sample containers filled with 80% ethanol, and subsequently identified using standard taxonomic methods. Half of the collected *S. calcitrans* flies were dissected, and their heads and abdomens were placed into separate Eppendorf tubes in pools of five. The remaining *Stomoxys* specimens were left intact and stored in Eppendorf tubes, also in pools of five. Horseflies were individually stored in Eppendorf tubes. All tubes were filled with 80% ethanol for preservation. Half of the samples were sent to the FLI for RNA extraction and *H. bovis* analysis using qRT-PCR. The remaining samples were retained as backup at the FUB.

### Examination for lice and mite infestation

In September 2023, 25 animals were randomly selected and examined for ectoparasites due to an increasing incidence of pruritus and crust formation at the tail base within the herd. The forehead, neck and tail base of each animal were combed using a lice comb and inspected for the presence of ectoparasites, such as sucking and biting lice.

In addition, diagnostic skin scrapings were taken from the base of the tail. Half of the collected material was stored in Eppendorf tubes with 80% ethanol, while the other half was stored dry. The dry samples were examined under a stereomicroscope, and motile mites were transferred to Eppendorf tubes containing 80% ethanol. Half of these samples were sent to the FUB for species identification, while the other half was sent to the FLI for RNA extraction and BovHepV analysis.

A retrospective analysis revealed that seven of the 25 animals tested positive for *H. bovis* in October 2022. In October 2023, eight additional animals tested positive, all of which had been negative the previous year.

### RNA extraction and RT-PCR analysis

Dry swabs from the nasal mucosa, conjunctiva and oral mucosa, as well as rectal stool samples, were incubated and shaken for 30 min at room temperature in 1 ml of ZB5d cell culture medium with penicillin/streptomycin and amphotericin [54]. Mixed quarter-milk samples, serum, and EDTA blood were used directly for nucleic acid extraction. Arthropod samples were homogenised in 600 µl of cell culture medium and a 5 mm steel ball in the TissueLyser II (Qiagen, Hilden, Germany) for 3 min at 30 Hz. *Stomoxys*, horseflies, biting midges, *Simulium* and mosquitoes were pooled for RNA extraction. All other vectors were extracted and analyzed individually. The viral RNA was extracted from 100 µl of the liquid phase using the NucleoMagVET kit (Macherey-Nagel, Düren, Germany) on the semi-automated KingFisher platform (KingFisher Flex Magnetic Particle Processor, Thermo Fisher Scientific) and eluted into 100 µl of elution buffer. To verify the success of the RNA extraction, an internal control RNA (IC-2 RNA) was added during the extraction process.

The existing BovHepV-1- and BovHepV-2-specific PCR assays were used to analyze the various sample matrices and bovine samples in general [31]. As it was anticipated that only small amounts of viral RNA would be detectable in the vectors under investigation, a highly sensitive panBovHepV RT-qPCR assay was developed for the general screening of BovHepV RNA. The new panBovHepV RT-qPCR utilizes the highly conserved 5´-UTR (untranslated region) of BovHepV. When selecting the primers and probe, BovHepV sequences of genotypes 1 and 2 were used, including those publicly available at the time in 2023 and those generated in-house. The following oligonucleotides were combined for the panBovHepV-Mix11 assay: BoHepV-172-F (5´-GRC CTC YTC GGT AGA TTT GG-3´), BoHepV-284-R (5´-CAG CCG CAC CCA CRG ATC-3`) and BoHepV-237-FAM (5´-FAM-TCC ACA CAG CCT GAT AGG GTC CT-BHQ1-3´). The selected primers amplify a 113 bp fragment from the 5´UTR of all BovHepV, including both genotype 1 and genotype 2 strains. The sensitivity and specificity of the panBovHepV-Mix11 assay were verified by silico analyses and confirmed by comparative analyses of known BovHepV-positive and -negative bovine samples (Table S1).

The composition of the RT-qPCR reactions was similar to that of the previously published BovHepV-1 and BovHepV-2-specific RT-qPCR assays [31]. Briefly, the RT-qPCR reactions contained 1.25 µl RNase-free water, 6.25 µl 2x RT-PCR buffer, 0.5 µl RT-PCR enzyme mix, 1 µl panBovHepV primer probe mix 11 (10 pmol/µl primer-for, 10 pmol/µl primer-rev, 2.5 pmol/µl FAM probe, in 0.1x Tris-EDTA (pH = 8.0)), 1 µl EGFP mix1-HEX (2.5 pmol/µl primer-for, 2.5 pmol/µl primer-rev, 2.5 pmol/µl HEX probe, in 0.1x Tris-EDTA (pH = 8.0)) and 2.5 µl of template RNA. All RT-qPCRs were performed on the CFX 96 real-time PCR cycler (Bio-Rad, Hercules, CA, USA) using AgPath ID™ One-Step RT-PCR reagents from Applied Biosystems™ (Waltham, USA). The temperature profile used was 10 min at 45 °C, 10 min at 95 °C, followed by 45 cycles of 15 s at 95 °C, 30 s at 56 °C, and 30 s at 72 °C. Fluorescence values (FAM, HEX) were recorded during the annealing step. Samples were considered positive if the quantification cycle value (Cq) was < 45.

## Results

### Screening of different sample types for BovHepV RNA

In December 2020, two months after the initial identification of *H. bovis* various samples were collected from seven cows with the highest virus loads (BovHep-1 cq values: 28.75 to 30.65; BovHep-2 cq values: 29.37 to 33.54) and subsequently analyzed via real-time RT-PCR to assess possible transmission routes. All nasal, ocular and buccal swabs, as well as milk and faecal samples, tested negative for BovHepV, whereas serum and EDTA samples collected from the same animals tested positive. Since serum samples contained higher viral loads in comparison to EDTA samples, serum was selected for subsequent virus detection from other animals shown in Table 1.

**Table 1:** Comparison of viral load in serum and EDTA blood samples

Animal	Matrice	BovHep Mix 5 FAM	BovHep2 Mix 4 FAM
1	serum EDTA	**32.79** no Cq	no Cq no Cq
2	serum EDTA	**29.37** **32.64**	no Cq no Cq
3	serum EDTA	no Cq no Cq	**31.07** **32.66**
4	serum EDTA	**28.65** **30.47**	no Cq no Cq
5	serum EDTA	no Cq no Cq	**33.51** **35.48**
6	serum EDTA	**28.46** **32.77**	no Cq no Cq
7	serum EDTA	**35.18** **35.24**	no Cq no Cq

### Screening of serum samples from calves and young heifers

Twenty serum samples each from calves and young heifers all tested negative for *H. bovis* using RT-qPCR. Cultural analysis of the nasal swabs confirmed the presence of Bovine Respiratory Syncytial Virus (BRSV) and *Pasteurella multocida*.

### Screening of serum samples from a neighboring farm

Seventy-five blood samples collected as part of the annual BoHV-1 surveillance collected from a neighboring fattening farm, located approx. 550 m from the BovHepV-positive farm all tested negative for the presence of *H. bovis* by qPCR.

### Collection of arthropods

During the study period, UV-light traps primarily captured *Culicoides* biting midges (*n* = 9,428), followed by mosquitoes (*n* = 99), black flies (*Simuliidae*, *n* = 86) and stable flies (*S. calcitrans*, *n* = 14). Aerial sweep netting was successful for the collection of stable flies (*n* = 1,101), as well as a small number of biting midges (*n* = 6) and horseflies (*Tabanidae*, *n* = 3) (Table 2). An overview of the feeding status of the collected arthropod species is shown in Table 3. The majority of the mosquitoes were identified as culicine species; only four of the 99 specimens were identified as *Anopheles* spp. No sucking or biting lice were detected, but all 25 examined cows were found to be infested with *Chorioptes* mites. A detailed overview of the specimens collected using UV-light traps is shown in Table S2 and a detailed overview of species and their feeding status is shown in Table S3.

**Table 2:** Overview of arthropods collected using UV-light traps or aerial sweep nets

	collected with UV-light traps				collected with aerial sweep nets		
	Stomoxys	Mosquitoes	Biting midges	Simulium	Stomoxys	Horseflies	Biting midges
May 2022	1	3	504	-	10	-	3
June 2022	2	10	2,238	-	217	1	-
July 2022	3	59	3,605	1	303	2	1
August 2022	7	25	2,999	85	404	-	2
September 2022	1	2	82	-	167	-	-
Total number	**14**	**99**	**9**,**428**	**86**	**1**,**101**	**3**	**6**

**Table 3:** Overview of species and feeding status of the collected arthropods

Month	Arthropod Species	Blood-fed	Indeterminate	Unfed	Damaged
May 2022	*Stomoxys*	-	11	-	-
	Biting midges	40	433	34	-
	Culicine mosquitoes	-	3	-	-
June 2022	*Stomoxys*	45	174	-	1
	Biting midges	76	1794	368	-
	Culicine mosquitoes	-	10	-	-
	Horseflies	-	1	-	-
July 2022	*Stomoxys*	76	230	-	-
	Biting midges	98	2532	976	-
	Culicine mosquitoes	-	55	-	-
	Anopheline mosquitoes	-	4	-	-
	Horseflies	-	2	-	-
	*Simulium*	-	1	-	-
August 2022	*Stomoxys*	84	327	-	-
	Biting midges	61	2618	322	-
	Culicine mosquitoes	-	25	-	-
	*Simulium*	-	85	-	-
September 2022	*Stomoxys*	36	132	-	-
	Biting midges	4	62	16	-
	Culicine mosquitoes	-	2	-	-

### Screening of arthropod samples by RT-qPCR and concurrent detection of BovHepV in cattle

None of the analyzed arthropod samples (*n* = 727) tested positive for *H. bovis* by high-sensitive panBovHepV-Mix11. In contrast, analysis of the 246 blood samples collected during the mandatory BoHV-1 surveillance demonstrated ongoing circulation of BovHepV on the farm in 2023, with 83 animals testing positive for BovHepV-1 and nine animals testing positive for BovHepV-2. Compared with the previous two years of testing, an increase in infection prevalence was observed. In October 2022, 72 of the 245 animals tested positive for BovHepV 1 while seven out of 245 tested positive for BovHepV-2. In September 2021, 53 out of 264 animals were positive for virus BovHepV-1 and three out of 264 for virus BovHepV-2 [31]. Out of 246 cattle blood samples analyzed, 100 cattle were tested positive using the panBovHepV-Mix11. Additionally, 83 samples were positive in the BovHepV-1 Mix5, while 8 samples tested positive in the BovHepV-2 Mix4. In comparison, 10 samples were only positive in the panBovHepV Mix11, with all of them having a quantification cycle value (Cq) greater than 36. Notably, no sample was specifically positive for either BovHepV-1 or BovHepV-2 and negative for panBovHep-Mix11 PCR. Data shown in Table S1.

## Discussion

Nearly ten years after the first description of *H. bovis*, its transmission routes remain unknown. Because hematophagous arthropods, including ticks, mosquitoes and biting flies transmit various members of the *Flaviviridae* family [11, 29], vector-borne transmission of BovHepV is considered plausible. Arthropod-borne spread may occur either through biological transmission, in which the virus replicates within a competent vector, or through mechanical transmission, in which infectious material is passively transferred between hosts, for instance via contaminated mouthparts.

Mosquito-borne viruses within the *Orthoflavivirus* genus, such as West Nile virus (WNV) [14] and Japanese encephalitis virus [47], illustrate the importance of biological vector competence within the *Flaviviridae* family. In addition, mechanical transmission of WNV by *S. calcitrans* has been demonstrated under experimental conditions [22], and the interrupted feeding behavior of stable flies may facilitate blood-borne transmission [57]. However, evidence for arthropod involvement in hepacivirus transmission remains scarce. Jogalong virus, a hepacivirus related to duck hepacivirus, was detected in a single *Culex annulirostris* mosquito in Australia, although this likely reflected residual virus from a prior blood meal rather than replication within the mosquito [67]. Similarly, *H. equi* RNA was not detected in any of the 5,338 mosquitoes, grouped into 566 pools, collected across Austria [3].

In the present study, none of the analyzed arthropod samples tested positive for BovHepV RNA and so these findings do not support a major role for mosquitoes, biting midges or stable flies in *H. bovis* transmission within the investigated herd. However, several limitations must be considered. First, the number of collected mosquitoes was relatively low, limiting the ability to detect rare infection events. Second, pooled sample analysis may reduce sensitivity when viral loads are low, particularly if only a single infected specimen is present in a pool [64]. Although no Minimum Infection Rate (MIR) could be calculated due to the absence of positive pools, the sample size nevertheless allows exclusion of high-prevalence vector infection under the study conditions. In our initial study, the prevalence of BovHepV-1 and BovHepV-2 was 24.6% (95% confidence interval [CI]: 19.2–30.6%) and 11.3% (95% CI: 7.5–16.1%), respectively [31]. Compared with other arthropod-borne infections such as bluetongue virus (BTV), for which within-herd seroprevalence has been reported to range from 96.2% to 100% (95% CI) [9], or Schmallenberg virus (SBV), with a within-herd seroprevalence of 86.3% (95% CI: 84.75–87.71) [42], the seroprevalence observed in our study was considerably lower. The prevalence of BTV, SBV, but also for WNV, and Rift Valley fever virus (RVFV) in their respective vectors varies considerably depending on environmental conditions, climatic factors, vector species composition, and geographic distribution. Reported prevalences for BTV range from 0.5% to 37.8% [10, 30, 33, 63], while SBV prevalence ranges from 0.25% to 18.6% [10, 25, 37, 52, 53, 70]. WNV prevalence in vectors has been reported to be between 0.07% and 9.2% [1], while RVFV prevalence in mosquitoes has been observed at levels up to 25% [24]. In contrast, none of the arthropod samples investigated in the present study tested positive for *H. bovis*. Given the high sensitivity of the applied pan-Hepacivirus-PCR, which would likely have detected low-prevalence infections like those reported for WNV or SBV, these findings strongly suggest the absence of viral RNA in the examined insects, indicating that they are unlikely to play a role as vectors. However, low-prevalence infections or sporadic mechanical transmission events cannot be completely ruled out. Importantly, PCR-based detection primarily assesses the presence of viral RNA and does not directly evaluate vector competence or short-term contamination of mouthparts immediately after feeding. Arthropod specimens were preserved in 80% ethanol and previous studies, including two that specifically used 80% ethanol and successfully detected viral RNA in insects [18, 70], have demonstrated reliable RT-PCR detection of arboviral RNA from hematophagous insects stored in ethanol concentrations ranging from 70% to 99.5% [34, 62, 65], indicating that the use of 80% ethanol in the present study is unlikely to have compromised viral RNA detectability.

Horseflies were collected only in small numbers, preventing meaningful assessment of their role. As recognized mechanical vectors of several livestock pathogens [20, 27, 28, 32], their potential involvement cannot be excluded and would require targeted investigation. Tick-borne transmission was considered unlikely in this specific herd because all animals were housed indoors without pasture access and no tick infestation was observed. Although *H. bovis* has previously been detected in *Rhipicephalus microplus* ticks collected from cattle in China [59, 69], this finding may reflect residual blood rather than replication within ticks.

In September 2023, the herd exhibited increasing signs of itching and crust formation at the base of the tail. Twenty-five animals were preselected based on clinical signs of mange and examined for the presence of ectoparasites. These animals were retrospectively matched with records of individuals previously identified as infected with BovHepV. Seven of twenty-five animals tested positive for *H. bovis* in October 2022. Eight more animals tested positive in October 2023 but had been negative the previous year. The identified *Chorioptes* mites tested negative for the BovHepV genome. *Chorioptes* mites acquire nutrients by superficially scraping the host’s skin with their chelicerae and absorbing tissue fluids, a feeding process that leads to irritation and pruritus in affected animals [46]. As these mites live on the skin and are non-burrowing [38] it seems unlikely that they are involved in the transmission of BovHepV, even if the number of samples was limited.

Collectively, these data provide no evidence that the investigated arthropods play a major role in BovHepV transmission within this herd. The additional finding that animals from a neighboring farm within the flight range of stable flies, mosquitoes and biting midges tested negative supports the absence of frequent vector-mediated spread under local conditions, although this observation alone does not exclude occasional transmission events.

Evidence for vertical transmission remains limited. While BovHepV RNA was previously detected in pooled calf sera [55], none of the calves or young heifers examined in the present study tested positive. These findings argue against frequent vertical transmission in this herd, but do not exclude rare events or transient perinatal infection. Longitudinal studies of well-characterized dam-calf pairs, ideally including sampling at birth before colostrum intake, would be required to conclusively address this question. This would be analogous to bovine viral diarrhea virus (BVDV), where fetal immune tolerance during the first trimester of gestation can lead to lifelong virus persistence [43].

Given the continued circulation of *H. bovis* within the herd and the occurrence of newly infected animals over multiple years, alternative transmission routes must be considered. The exclusive detection of viral RNA in blood, but not in other matrices, supports the hypothesis of blood-associated transmission. Analogous to the related HCV in humans, transmission through direct blood contact is biologically plausible [44]. Iatrogenic spread via reuse of needles during vaccination or intravenous treatments has been previously proposed [4, 26] and is consistent with the known mechanical transmission of other bovine viruses, such as BTV, through shared needles [16]. Husbandry procedures associated with potential blood exposure, such as hoof trimming, should also be evaluated as possible risk factors. Experimental studies in horses have demonstrated successful transmission of *H. equi* via plasma or serum transfusion [48, 51], further supporting blood-borne transmission as a biologically plausible pathway for BovHepV. Sexual transmission, for instance via contaminated semen or embryo transfer equipment, also warrants consideration [26]. However, this route appears unlikely in this case, given the negative findings in all calves and heifers.

In conclusion, our data provides no evidence supporting a major role for the investigated arthropods or frequent vertical transmission in *H. bovis* spread within this dairy herd. The findings instead favor blood-associated transmission as the most plausible mechanism under the conditions studied. Therefore, management practices should be considered when evaluating disease prevention strategies. Effective farm management of cattle herds is essential to minimize infection risks from blood contact, such as that caused by contaminated needles or syringes used during routine procedures. Nevertheless, rare vector-mediated events, low-prevalence infections, or additional transmission pathways cannot be definitively excluded and should be addressed in future experimental and epidemiological studies.

## Supplementary Information

Below is the link to the electronic supplementary material.

Supplementary Material 1 (PDF 352 KB)

## Data Availability

All data generated or analysed during this study are included in this published article and its supplementary information files.

## References

[1] Abbas I, Ahmed F, Muqaddas H, Alberti A, Varcasia A, Sedda L (2025) Epidemiology and surveillance of West Nile virus in the Mediterranean Basin during 2010–2023: A systematic review. Curr Res Parasitol Vector Borne Dis 7:100277. 10.1016/j.crpvbd.2025.100277 PMC12205625 40586111

[2] Anggakusuma, Brown RJP, Banda DH, Todt D, Vieyres G, Steinmann E, Pietschmann T (2016) Hepacivirus NS3/4A proteases interfere with MAVS signaling in both their cognate animal hosts and humans: Implications for zoonotic transmission. J Virol 90:10670–10681. 10.1128/JVI.01634-16 PMC5110154 27654291

[3] Badenhorst M, de Heus P, Auer A, Rumenapf T, Tegtmeyer B, Kolodziejek J, Nowotny N, Steinmann E, Cavalleri JV (2019) No evidence of mosquito involvement in the transmission of equine hepacivirus (Flaviviridae) in an epidemiological survey of Austrian horses. Viruses. 10.3390/v11111014 PMC6893842 31683893

[4] Baechlein C, Fischer N, Grundhoff A, Alawi M, Indenbirken D, Postel A, Baron AL, Offinger J, Becker K, Beineke A, Rehage J, Becher P (2015) Identification of a novel hepacivirus in domestic cattle from Germany. J Virol 89:7007–7015. 10.1128/JVI.00534-15 PMC4473572 25926652

[5] Baechlein C, Baron AL, Meyer D, Gorriz-Martin L, Pfankuche VM, Baumgartner W, Polywka S, Peine S, Fischer N, Rehage J, Becher P (2019) Further characterization of bovine hepacivirus: Antibody response, course of infection, and host tropism. Transbound Emerg Dis 66:195–206. 10.1111/tbed.12999 30126081

[6] Blitvich BJ, Firth AE (2015) Insect-specific flaviviruses: a systematic review of their discovery, host range, mode of transmission, superinfection exclusion potential and genomic organization. Viruses 7:1927–1959. 10.3390/v7041927 PMC4411683 25866904

[7] Burbelo PD, Dubovi EJ, Simmonds P, Medina JL, Henriquez JA, Mishra N, Wagner J, Tokarz R, Cullen JM, Iadarola MJ, Rice CM, Lipkin WI, Kapoor A (2012) Serology-enabled discovery of genetically diverse hepaciviruses in a new host. J Virol 86:6171–6178. 10.1128/JVI.00250-12 PMC3372197 22491452

[8] Canal CW, Weber MN, Cibulski SP, Silva MS, Puhl DE, Stalder H, Peterhans E (2017) A novel genetic group of bovine hepacivirus in archival serum samples from Brazilian cattle. Biomed Res Int 2017:4732520. 10.1155/2017/4732520 PMC5585675 28904959

[9] Cargnel M, Simons X, De Leeuw I, De Regge N, Hanon JB (2026) High seroprevalence of bluetongue virus serotype 3 in Belgian cattle and sheep after the 2024 epidemic. Viruses. 10.3390/v18030396 PMC13030625 41902304

[10] Catania AM, Grego E, Tomassone L, Giulia C, Giorgia DM, Sara F, D’Angelo A (2025) Silent circulation of Bluetongue and Schmallenberg viruses in *Culicoides* biting midges in northwestern Italy: implications for the 2024 BTV outbreak. J Med Entomol 62:1582–1589. 10.1093/jme/tjaf128 PMC12616229 41060278

[11] Chala B, Hamde F (2021) Emerging and re-emerging vector-borne infectious diseases and the challenges for control: A review. Front Public Health 9:715759. 10.3389/fpubh.2021.715759 PMC8524040 34676194

[12] Chang WS, Eden JS, Hartley WJ, Shi M, Rose K, Holmes EC (2019) Metagenomic discovery and co-infection of diverse wobbly possum disease viruses and a novel hepacivirus in Australian brushtail possums. One Health Outlook 1:5. 10.1186/s42522-019-0006-x PMC7990097 33829126

[13] Chu L, Jin M, Feng C, Wang X, Zhang D (2019) A highly divergent hepacivirus-like flavivirus in domestic ducks. J Gen Virol 100:1234–1240. 10.1099/jgv.0.001298 31282853

[14] Ciota AT (2017) West Nile virus and its vectors. Curr Opin Insect Sci 22:28–36. 10.1016/j.cois.2017.05.002 28805636

[15] Corman VM, Grundhoff A, Baechlein C, Fischer N, Gmyl A, Wollny R, Dei D, Ritz D, Binger T, Adankwah E, Marfo KS, Annison L, Annan A, Adu-Sarkodie Y, Oppong S, Becher P, Drosten C, Drexler JF (2015) Highly divergent hepaciviruses from African cattle. J Virol 89:5876–5882. 10.1128/JVI.00393-15 PMC4442428 25787289

[16] Darpel KE, Barber J, Hope A, Wilson AJ, Gubbins S, Henstock M, Frost L, Batten C, Veronesi E, Moffat K, Carpenter S, Oura C, Mellor PS, Mertens PP (2016) Using shared needles for subcutaneous inoculation can transmit bluetongue virus mechanically between ruminant hosts. Sci Rep 6:20627. 10.1038/srep20627 PMC4745043 26853457

[17] de Martinis C, Cardillo L, Esposito C, Viscardi M, Barca L, Cavallo S, D’Alessio N, Martella V, Fusco G (2022) First identification of bovine hepacivirus in wild boars. Sci Rep 12:11678. 10.1038/s41598-022-15928-7 PMC9270363 35804025

[18] De Regge N, Deblauwe I, De Deken R, Vantieghem P, Madder M, Geysen D, Smeets F, Losson B, van den Berg T, Cay AB (2012) Detection of Schmallenberg virus in different *Culicoides* spp. by real-time RT-PCR. Transbound Emerg Dis 59:471–475. 10.1111/tbed.12000 23025501

[19] Deng Y, Guan SH, Wang S, Hao G, Rasmussen TB (2018) The detection and phylogenetic analysis of bovine hepacivirus in China. Biomed Res Int 2018:6216853. 10.1155/2018/6216853 PMC6000834 29955606

[20] Desquesnes M, Biteau-Coroller F, Bouyer J, Dia ML, Foil L (2009) Development of a mathematical model for mechanical transmission of trypanosomes and other pathogens of cattle transmitted by tabanids. Int J Parasitol 39:333–346. 10.1016/j.ijpara.2008.07.004 18755195

[21] Dodge HR (1953) Identifying common flies. Public Health Rep 1896:68:345–350 PMC2023985 13037962

[22] Doyle MS, Swope BN, Hogsette JA, Burkhalter KL, Savage HM, Nasci RS (2011) Vector competence of the stable fly (Diptera: Muscidae) for West Nile virus. J Med Entomol 48:656–668. 10.1603/me10167 21661328

[23] Drexler JF, Corman VM, Muller MA, Lukashev AN, Gmyl A, Coutard B, Adam A, Ritz D, Leijten LM, van Riel D, Kallies R, Klose SM, Gloza-Rausch F, Binger T, Annan A, Adu-Sarkodie Y, Oppong S, Bourgarel M, Rupp D, Hoffmann B, Schlegel M, Kummerer BM, Kruger DH, Schmidt-Chanasit J, Setien AA, Cottontail VM, Hemachudha T, Wacharapluesadee S, Osterrieder K, Bartenschlager R, Matthee S, Beer M, Kuiken T, Reusken C, Leroy EM, Ulrich RG, Drosten C (2013) Evidence for novel hepaciviruses in rodents. PLoS Pathog 9:e1003438. 10.1371/journal.ppat.1003438 PMC3688547 23818848

[24] Ebogo-Belobo JT, Kenmoe S, Abanda NN, Bowo-Ngandji A, Mbaga DS, Magoudjou-Pekam JN, Kame-Ngasse GI, Tchatchouang S, Menkem EZ, Okobalemba EA, Noura EA, Meta-Djomsi D, Maïdadi-Foudi M, Kenfack-Zanguim J, Kenfack-Momo R, Kengne-Nde C, Esemu SN, Mbacham WF, Sadeuh-Mba SA, Ndip L, Njouom R (2023) Contemporary epidemiological data of Rift Valley fever virus in humans, mosquitoes and other animal species in Africa: A systematic review and meta-analysis. Vet Med Sci 9:2309–2328. 10.1002/vms3.1238 PMC10508527 37548116

[25] Elbers AR, Meiswinkel R, van Weezep E, van Sloet MM, Kooi EA (2013) Schmallenberg virus in *Culicoides* spp. biting midges, the Netherlands, 2011. Emerg Infect Dis 19:106–109. 10.3201/eid1901.121054 PMC3558002 23260040

[26] Elia G, Caringella F, Lanave G, Martella V, Losurdo M, Tittarelli M, Colitti B, Decaro N, Buonavoglia C (2020) Genetic heterogeneity of bovine hepacivirus in Italy. Transbound Emerg Dis 67:2731–2740. 10.1111/tbed.13628 32426936

[27] Foil LD (1989) Tabanids as vectors of disease agents. Parasitol Today 5:88–96. 10.1016/0169-4758(89)90009-4 15463186

[28] Foil LD, Adams WV, McManus JM, Issel CJ (1987) Bloodmeal residues on mouthparts of *Tabanus fuscicostatus* (Diptera: Tabanidae) and the potential for mechanical transmission of pathogens. J Med Entomol 24:613–616. 10.1093/jmedent/24.6.613 2826787

[29] Fuente Jdl, Estrada-Pena A, Venzal JM, Kocan KM, Sonenshine DE (2008) Overview: Ticks as vectors of pathogens that cause disease in humans and animals. FBL 13:6938–6946. 10.2741/3200 18508706

[30] Goffredo M, Catalani M, Federici V, Portanti O, Marini V, Mancini G, Quaglia M, Santilli A, Teodori L, Savini G (2015) Vector species of *Culicoides* midges implicated in the 2012–2014 Bluetongue epidemics in Italy. Vet Ital 51:131–138. 10.12834/VetIt.771.3854.1 26129664

[31] Hake N, von Holtum C, Höper D, Nijhof AM, Dietze K, Hoffmann B (2026) Identification and long-term detection of hepacivirus bovis genotype 1 and 2 on a cattle farm in Germany. Viruses 18:78 10.3390/v18010078 PMC12846713 41600842

[32] Hocking B (1971) Blood-sucking behavior of terrestrial arthropods. Annu Rev Entomol 16:1–26. 10.1146/annurev.en.16.010171.000245 4928343

[33] Hoffmann B, Bauer B, Bauer C, Bätza HJ, Beer M, Clausen PH, Geier M, Gethmann JM, Kiel E, Liebisch G, Liebisch A, Mehlhorn H, Schaub GA, Werner D, Conraths FJ (2009) Monitoring of putative vectors of bluetongue virus serotype 8, Germany. Emerg Infect Dis 15:1481–1484. 10.3201/eid1509.090562 PMC2819873 19788820

[34] Kai I, Kobayashi D, Itokawa K, Sanjoba C, Itoyama K, Isawa H (2024) Evaluation of long-term preservation methods for viral RNA in mosquitoes at room temperature. J Virol Methods 325:114887. 10.1016/j.jviromet.2024.114887 38237867

[35] Kapoor A, Simmonds P, Gerold G, Qaisar N, Jain K, Henriquez JA, Firth C, Hirschberg DL, Rice CM, Shields S, Lipkin WI (2011) Characterization of a canine homolog of hepatitis C virus. Proc Natl Acad Sci U S A 108:11608–11613. 10.1073/pnas.1101794108 PMC3136326 21610165

[36] Kapoor A, Simmonds P, Scheel TK, Hjelle B, Cullen JM, Burbelo PD, Chauhan LV, Duraisamy R, Sanchez Leon M, Jain K, Vandegrift KJ, Calisher CH, Rice CM, Lipkin WI (2013) Identification of rodent homologs of hepatitis C virus and pegiviruses. mBio 4:e00216–e00213. 10.1128/mBio.00216-13 PMC3622934 23572554

[37] Kęsik-Maliszewska J, Larska M, Collins ÁB, Rola J (2019) Post-epidemic distribution of Schmallenberg virus in *Culicoides* arbovirus vectors in Poland. Viruses. 10.3390/v11050447 PMC6563501 31100887

[38] Ketzis JK (2023) Mange in Cattle. MSD Veterinary Manual, MSD Veterinary Manual

[39] Linnen J, Wages J Jr., Zhang-Keck ZY, Fry KE, Krawczynski KZ, Alter H, Koonin E, Gallagher M, Alter M, Hadziyannis S, Karayiannis P, Fung K, Nakatsuji Y, Shih JW, Young L, Piatak M Jr., Hoover C, Fernandez J, Chen S, Zou JC, Morris T, Hyams KC, Ismay S, Lifson JD, Hess G, Foung SK, Thomas H, Bradley D, Margolis H, Kim JP (1996) Molecular cloning and disease association of hepatitis G virus: a transfusion-transmissible agent. Science 271:505–508. 10.1126/science.271.5248.505 8560265

[40] Lu G, Jia K, Ping X, Huang J, Luo A, Wu P, Li S (2018) Novel bovine hepacivirus in dairy cattle, China. Emerg Microbes Infect 7:54. 10.1038/s41426-018-0055-8 PMC5883034 29615608

[41] Lyons S, Kapoor A, Schneider BS, Wolfe ND, Culshaw G, Corcoran B, Durham AE, Burden F, McGorum BC, Simmonds P (2014) Viraemic frequencies and seroprevalence of non-primate hepacivirus and equine pegiviruses in horses and other mammalian species. J Gen Virol 95:1701–1711. 10.1099/vir.0.065094-0 24814924

[42] Méroc E, Poskin A, Van Loo H, Quinet C, Van Driessche E, Delooz L, Behaeghel I, Riocreux F, Hooyberghs J, De Regge N, Caij AB, van den Berg T, van der Stede Y (2013) Large-scale cross-sectional serological survey of Schmallenberg virus in Belgian cattle at the end of the first vector season. Transbound Emerg Dis 60:4–8. 10.1111/tbed.12042 23206240

[43] Moennig V, Liess B (1995) Pathogenesis of intrauterine infections with bovine viral diarrhea virus. Veterinary Clin North America: Food Anim Pract 11:477–487. 10.1016/S0749-0720(15)30462-X 8581858

[44] Moosavy SH, Davoodian P, Nazarnezhad MA, Nejatizaheh A, Eftekhar E, Mahboobi H (2017) Epidemiology, transmission, diagnosis, and outcome of Hepatitis C virus infection. Electron Physician 9:5646–5656. 10.19082/5646 PMC5718874 29238510

[45] Nerurkar VR, Chua PK, Hoffmann PR, Dashwood W-M, Shikuma CM, Yanagihara R (1998) High prevalence of GB virus C/hepatitis G virus infection among homosexual men infected with human immunodeficiency virus type 1: Evidence for sexual transmission. J Med Virol 56:123–127. 10.1002/(sici)1096-9071(199810)56:2<123::Aid-jmv4>3.0.Co;2-a 9746067

[46] Oconnor BM (2009) Chap. 169 - Mites. In: Resh VH, Cardé RT (eds) Encyclopedia of Insects (Second Edition). Academic Press, San Diego, pp 643–649

[47] Oliveira ARS, Strathe E, Etcheverry L, Cohnstaedt LW, McVey DS, Piaggio J, Cernicchiaro N (2018) Assessment of data on vector and host competence for Japanese encephalitis virus: A systematic review of the literature. Prev Vet Med 154:71–89. 10.1016/j.prevetmed.2018.03.018 29685447

[48] Pfaender S, Walter S, Grabski E, Todt D, Bruening J, Romero-Brey I, Gather T, Brown RJ, Hahn K, Puff C, Pfankuche VM, Hansmann F, Postel A, Becher P, Thiel V, Kalinke U, Wagner B, Bartenschlager R, Baumgartner W, Feige K, Pietschmann T, Cavalleri JM, Steinmann E (2017) Immune protection against reinfection with nonprimate hepacivirus. Proc Natl Acad Sci U S A 114:E2430–E2439. 10.1073/pnas.1619380114 PMC5373355 28275093

[49] Postler TS, Beer M, Blitvich BJ, Bukh J, de Lamballerie X, Drexler JF, Imrie A, Kapoor A, Karganova GG, Lemey P, Lohmann V, Simmonds P, Smith DB, Stapleton JT, Kuhn JH (2023) Renaming of the genus Flavivirus to Orthoflavivirus and extension of binomial species names within the family Flaviviridae. Arch Virol 168:224. 10.1007/s00705-023-05835-1 37561168

[50] Quan PL, Firth C, Conte JM, Williams SH, Zambrana-Torrelio CM, Anthony SJ, Ellison JA, Gilbert AT, Kuzmin IV, Niezgoda M, Osinubi MO, Recuenco S, Markotter W, Breiman RF, Kalemba L, Malekani J, Lindblade KA, Rostal MK, Ojeda-Flores R, Suzan G, Davis LB, Blau DM, Ogunkoya AB, Alvarez Castillo DA, Moran D, Ngam S, Akaibe D, Agwanda B, Briese T, Epstein JH, Daszak P, Rupprecht CE, Holmes EC, Lipkin WI (2013) Bats are a major natural reservoir for hepaciviruses and pegiviruses. Proc Natl Acad Sci U S A 110:8194–8199. 10.1073/pnas.1303037110 PMC3657805 23610427

[51] Ramsay JD, Evanoff R, Wilkinson TE Jr., Divers TJ, Knowles DP, Mealey RH (2015) Experimental transmission of equine hepacivirus in horses as a model for hepatitis C virus. Hepatology 61:1533–1546. 10.1002/hep.27689 25580897

[52] Rasmussen LD, Kristensen B, Kirkeby C, Rasmussen TB, Belsham GJ, Bødker R, Bøtner A (2012) Culicoids as vectors of Schmallenberg virus. Emerg Infect Dis 18:1204–1206. 10.3201/eid1807.120385 PMC3376822 22709978

[53] Rasmussen LD, Kirkeby C, Bødker R, Kristensen B, Rasmussen TB, Belsham GJ, Bøtner A (2014) Rapid spread of Schmallenberg virus-infected biting midges (*Culicoides* spp.) across Denmark in 2012. Transbound Emerg Dis 61:12–16. 10.1111/tbed.12189 24219171

[54] Rau J, Werner D, Beer M, Hoper D, Kampen H (2022) The microbial RNA metagenome of *Aedes albopictus* (Diptera: Culicidae) from Germany. Parasitol Res 121:2587–2599. 10.1007/s00436-022-07576-7 PMC9378336 35857094

[55] Sadeghi M, Kapusinszky B, Yugo DM, Phan TG, Deng X, Kanevsky I, Opriessnig T, Woolums AR, Hurley DJ, Meng XJ, Delwart E (2017) Virome of US bovine calf serum. Biologicals 46:64–67. 10.1016/j.biologicals.2016.12.009 PMC5654489 28100412

[56] Schmidt B, Korn K, Fleckenstein B (1996) Molecular evidence for transmission of hepatitis G virus by blood transfusion. Lancet 347:909. 10.1016/s0140-6736(96)91396-3 8622433

[57] Schofield S, Torr SJ (2002) A comparison of the feeding behaviour of tsetse and stable flies. Med Vet Entomol 16:177–185. 10.1046/j.1365-2915.2002.00361.x 12109712

[58] Service MW, Service M (2012) Medical entomology for students. Cambridge University Press

[59] Shao JW, Guo LY, Yuan YX, Ma J, Chen JM, Liu Q (2021) A novel subtype of bovine hepacivirus identified in ticks reveals the genetic diversity and evolution of bovine hepacivirus. Viruses. 10.3390/v13112206 PMC8623979 34835012

[60] Shi M, Lin XD, Vasilakis N, Tian JH, Li CX, Chen LJ, Eastwood G, Diao XN, Chen MH, Chen X, Qin XC, Widen SG, Wood TG, Tesh RB, Xu J, Holmes EC, Zhang YZ (2016) Divergent viruses discovered in arthropods and vertebrates revise the evolutionary history of the Flaviviridae and related viruses. J Virol 90:659–669. 10.1128/JVI.02036-15 PMC4702705 26491167

[61] Stark K, Bienzle U, Hess G, Engel AM, Hegenscheid B, Schluter V (1996) Detection of the hepatitis G virus genome among injecting drug users, homosexual and bisexual men, and blood donors. J Infect Dis 174:1320–1323. 10.1093/infdis/174.6.1320 8940225

[62] Uiterwijk M, Jacobs F, de Boer K, Feddema AJ, Vloet RPM, Dik M, Gonzales JL, van Rijn PA, Elbers ARW, Holwerda M (2025) *Culicoides* species involved in the BTV-3 epidemic, the Netherlands, 2023–2024. Med Vet Entomol. 10.1111/mve.70007 PMC12865740 40810503

[63] Vanbinst T, Vandenbussche F, Vandemeulebroucke E, De Leeuw I, Deblauwe I, De Deken G, Madder M, Haubruge E, Losson B, De Clercq K (2009) Bluetongue virus detection by real-time RT-PCR in *Culicoides* captured during the 2006 epizootic in Belgium and development of an internal control. Transbound Emerg Dis 56:170–177. 10.1111/j.1865-1682.2009.01077.x 19432638

[64] Vandenbussche F, Vanbinst T, Vandemeulebroucke E, Goris N, Sailleau C, Zientara S, De Clercq K (2008) Effect of pooling and multiplexing on the detection of bluetongue virus RNA by real-time RT-PCR. J Virol Methods 152:13–17. 10.1016/j.jviromet.2008.06.005 18590769

[65] Voigt A, Kampen H, Heuser E, Zeiske S, Hoffmann B, Höper D, Holsteg M, Sick F, Ziegler S, Wernike K, Beer M, Werner D (2024) Bluetongue virus serotype 3 and Schmallenberg virus in *Culicoides* biting midges, western Germany, 2023. Emerg Infect Dis 30:1438–1441. 10.3201/eid3007.240275 PMC11210651 38916645

[66] Walter S, Rasche A, Moreira-Soto A, Pfaender S, Bletsa M, Corman VM, Aguilar-Setien A, Garcia-Lacy F, Hans A, Todt D, Schuler G, Shnaiderman-Torban A, Steinman A, Roncoroni C, Veneziano V, Rusenova N, Sandev N, Rusenov A, Zapryanova D, Garcia-Bocanegra I, Jores J, Carluccio A, Veronesi MC, Cavalleri JMV, Drosten C, Lemey P, Steinmann E, Drexler JF (2017) Differential infection patterns and recent evolutionary origins of equine hepaciviruses in donkeys. J Virol. 10.1128/JVI.01711-16 PMC5165184 27795428

[67] Williams SH, Levy A, Yates RA, Somaweera N, Neville PJ, Nicholson J, Lindsay MDA, Mackenzie JS, Jain K, Imrie A, Smith DW, Lipkin WI (2020) Discovery of Jogalong virus, a novel hepacivirus identified in a *Culex annulirostris* (Skuse) mosquito from the Kimberley region of Western Australia. PLoS ONE 15:e0227114. 10.1371/journal.pone.0227114 PMC6941808 31899786

[68] Workman AM, Harhay GP, Groves JT, Vander Ley BL (2024) Two bovine hepacivirus genome sequences from U.S. cattle. J Vet Diagn Invest 36:274–277. 10.1177/10406387231225656 PMC10929628 38414254

[69] Yuan S, Yao XY, Lian CY, Kong S, Shao JW, Zhang XL (2023) Molecular detection and genetic characterization of bovine hepacivirus identified in ticks collected from cattle in Harbin, northeastern China. Front Vet Sci 10:1093898. 10.3389/fvets.2023.1093898 PMC10016144 36937022

[70] Zeiske S, Kampen H, Sick F, Dähn O, Voigt A, Heuser E, Beer M, Werner D, Wernike K (2025) Monitoring of Schmallenberg virus, bluetongue virus and epizootic haemorrhagic disease virus in biting midges in Germany 2019–2023. Parasit Vectors 18:262. 10.1186/s13071-025-06913-w PMC12229043 40618098

