# Supplementary material for: Lack of evidence for arthropod or vertical transmission of Hepacivirus bovis in a persistently infected dairy herd
Source: Arch Virol. 2026 Sep 28;171(10):286. doi: 10.1007/s00705-026-06747-6 (PMC13619772; doi:10.1007/s00705-026-06747-6)
Supplement: Supplementary file 1 [file 705_2026_6747_MOESM1_ESM.pdf]

Supplementary Materials

Table S1: Comparison of the results from the herd survey conducted in October 2023 (n=246), which were tested with the panBovHep-Mix11 and the specific PCRs for BovHepV-1 and -2 [30]. *

Sample No	panBovHep Mix11	BovHep1 Mix5	BovHep2 Mix4
BH 37/23_01	26,67	no Cq	31,96
BH 37/23_03	27,34	31,05	no Cq
BH 37/23_04	37,32	no Cq	no Cq
BH 37/23_06	23,47	28,68	no Cq
BH 37/23_07	30,73	34,57	no Cq
BH 37/23_10	25,21	30,03	no Cq
BH 37/23_13	33,57	37,59	no Cq
BH 37/23_14	26,48	28,49	no Cq
BH 37/23_16	26,45	30,58	no Cq
BH 37/23_17	35,87	no Cq	no Cq
BH 37/23_18	27,93	31,14	no Cq
BH 37/23_19	26,4	29,98	no Cq
BH 37/23_20	27,18	30,79	no Cq
BH 37/23_21	36,12	no Cq	no Cq
BH 37/23_26	24,27	28,01	no Cq
BH 37/23_27	25,12	29,16	no Cq
BH 37/23_29	25,5	27,04	no Cq
BH 37/23_30	36,06	39,37	no Cq
BH 37/23_34	25,91	no Cq	32,03
BH 37/23_38	33,89	35,8	no Cq
BH 37/23_39	25,2	26,96	no Cq
BH 37/23_40	25,32	30,92	no Cq
BH 37/23_41	28,32	32,65	no Cq
BH 37/23_50	34,14	36,83	no Cq
BH 37/23_51	26,25	35,47	no Cq
BH 37/23_53	37,53	no Cq	no Cq
BH 37/23_56	29,09	32,56	no Cq
BH 37/23_58	29,98	33,02	no Cq
BH 37/23_60	32,6	36,27	no Cq
BH 37/23_63	36,46	39,61	no Cq
BH 37/23_64	25,43	29,83	no Cq
BH 37/23_65	23,39	25,02	no Cq
BH 37/23_66	27,08	32,58	no Cq
BH 37/23_69	27,48	no Cq	33,03
BH 37/23_70	31,46	39,63	no Cq
BH 37/23_73	34,12	36,31	no Cq
BH 37/23_74	26,67	29,33	no Cq
BH 37/23_75	32,28	34,71	no Cq
BH 37/23_78	31,39	34,58	no Cq
BH 37/23_82	32,91	40,39	no Cq
BH 37/23_83	39,27	no Cq	no Cq
BH 37/23_84	29,93	35,85	no Cq
BH 37/23_85	20,8	23,44	no Cq
BH 37/23_87	25,28	30,25	no Cq

BH 37/23_92	26,37	29,62	no Cq
BH 37/23_93	28,63	30,82	no Cq
BH 37/23_94	25,02	28,72	no Cq
BH 37/23_103	27,96	31,16	no Cq
BH 37/23_105	28,33	29,3	no Cq
BH 37/23_106	25,84	29,06	no Cq
BH 37/23_107	24,05	25,28	no Cq
BH 37/23_109	38,18	no Cq	no Cq
BH 37/23_110	26,31	29,28	no Cq
BH 37/23_111	29,93	34,31	no Cq
BH 37/23_113	29,4	no Cq	37,77
BH 37/23_115	31,27	34,19	no Cq
BH 37/23_116	31,66	34,03	no Cq
BH 37/23_118	28,15	no Cq	35,25
BH 37/23_121	38,27	no Cq	no Cq
BH 37/23_126	27,62	31,59	no Cq
BH 37/23_128	29,19	33,71	no Cq
BH 37/23_130	24,32	27,79	no Cq
BH 37/23_131	28,22	no Cq	34,75
BH 37/23_132	27,25	28,64	no Cq
BH 37/23_141	29,04	31,74	no Cq
BH 37/23_142	38,31	no Cq	no Cq
BH 37/23_144	25,26	28,33	no Cq
BH 37/23_147	26,32	30,71	no Cq
BH 37/23_148	23,73	27,16	no Cq
BH 37/23_153	25,18	28,35	no Cq
BH 37/23_154	37,05	no Cq	no Cq
BH 37/23_157	25,24	26,62	no Cq
BH 37/23_158	25,87	28,93	no Cq
BH 37/23_159	25,81	31	no Cq
BH 37/23_161	25,34	26,95	no Cq
BH 37/23_163	27,71	30,67	no Cq
BH 37/23_164	23,46	28,14	no Cq
BH 37/23_168	22,41	27,48	no Cq
BH 37/23_172	25,71	29,24	no Cq
BH 37/23_181	26,2	25,15	no Cq
BH 37/23_189	29,12	32	no Cq
BH 37/23_191	24,48	24,91	no Cq
BH 37/23_201	33,24	34,87	no Cq
BH 37/23_206	29,74	33,61	no Cq
BH 37/23_207	27,25	28,17	no Cq
BH 37/23_208	32,3	34,18	no Cq
BH 37/23_209	26,67	27,22	no Cq
BH 37/23_210	21,47	20,72	no Cq
BH 37/23_212	38,57	no Cq	no Cq
BH 37/23_213	32,09	32,41	no Cq
BH 37/23_214	28,08	28,66	no Cq
BH 37/23_215	24,68	26,02	no Cq
BH 37/23_221	25,37	25,9	no Cq

BH 37/23_222	23,62	24,74	no Cq
BH 37/23_226	26,46	27,4	no Cq
BH 37/23_228	24,81	26,55	no Cq
BH 37/23_232	25,21	35,57	32,22
BH 37/23_235	25,22	no Cq	32,16
BH 37/23_236	26,35	29	no Cq
BH 37/23_244	21,21	22,16	no Cq

*Please note: Samples, with negative results in all three assays were not listed.

Table S2. Detailed overview of arthropods collected using UV-light traps

**Month**	**Trap location**	***Stomoxys***	**Culicine and anopheline mosquitoes**	**Biting midges**	***Simulium***
**May 2022**	Feed alley	1	1	130	-
	Walkway	-	-	330	-
	Outside	-	2	44	-
	Total number	**1**	**3**	**504**	**0**
**June 2022**	Feed alley	2	4	1,387	-
	Walkway	-	4	567	-
	Outside	-	2	284	-
	Total number	**2**	**10**	**2,238**	**0**
**July 2022**	Feed alley	1	20, incl. three *Anopheles* spp.	1,284	-
	Walkway	1	9	1,204	1
	Outside	1	30, including one *Anopheles* spp.	1,117	-
	Total number	**3**	**59**	**3,605**	**1**
**August 2022**	Feed alley	4	10	1,249	41
	Walkway	2	9	1,148	44
	Outside	1	6	604	-
	Total number	**7**	**25**	**2,999**	**85**
**September 2022**	Feed alley	1	-	41	-
	Walkway	--	-	37	-
	Outside		2	4	-
	Total number	**1**	**2**	**82**	**0**

Total number	14	99	9,428	86

Table S3. overview of species and feeding status

Month	Labeling on Eppendorf tubes	Trap location	Arthropod species	Fed	Uncertain	Unfed	Destroyed
May 2022	1 + 2	Aerial sweep net	*Stomoxys*	-	2 x 5	-	-
	3	Feed alley	*Stomoxys*	-	1	-	-
	4	Walkway	Biting midges	8	-	-	-
	5	Walkway	Biting midges	-	-	1 x 25	-
	6 - 16	Walkway	Biting midges	-	11 x 25	-	-
	17	Walkway	Biting midges	-	22	-	-
	18	Outside	Biting midges	-	1 x 25	-	-
	19	Outside	Biting midges	-	17	-	-
	20	Outside	Biting midges	-	-	2	-
	21	Feed alley	Biting midges	1 x 25	-	-	-
	22	Feed alley	Biting midges	7	-	-	-
	23	Feed alley	Biting midges	-	-	7	-
	24 - 26	Feed alley	Biting midges	-	3 x 25	-	-
	27	Feed alley	Biting midges	-	16	-	-
	28	Feed alley	Culicine mosquitoes	-	1	-	-
	29 + 30	Outside	Culicine mosquitoes	-	2 x 1	-	-
	31	Aerial sweep net	Biting midges	-	3	-	-

Month	Labeling on Eppendorf tubes	Trap location	Arthropod species	Fed	Uncertain	Unfed	Destroyed
June 2022	1	Aerial sweep net	*Stomoxys*	-	-	-	1 (abdomen missing)
	2 - 35	Aerial sweep net	*Stomoxys*	-	34 x 5	-	-
	36	Aerial sweep net	*Stomoxys*	-	2	-	-
	37 - 45	Aerial sweep net	*Stomoxys*	9 x 5	-	-	-
	46	Feed alley	*Stomoxys*	-	2	-	-
	1	Outside	Biting midges	9	-	-	-
	2	Outside	Biting midges	-	-	24	-
	3 - 12	Outside	Biting midges	-	10 x 25	-	-
	13	Outside	Biting midges	-	1	-	-
	14	Walkway	Biting midges	16	-	-	-
	15 - 17	Walkway	Biting midges	-	-	3 x 25	-
	18	Walkway	Biting midges	-	-	15	-
	19 - 36	Walkway	Biting midges	-	18 x 25	-	-
	37	Walkway	Biting midges	-	11	-	-
	38 - 39	Feed alley	Biting midges	2 x 25	-	-	-
	40	Feed alley	Biting midges	1	-	-	-
	41 - 50	Feed alley	Biting midges	-	-	10 x 25	-
	51	Feed alley	Biting midges	-	-	4	-
	52 - 94	Feed alley	Biting midges	-	43 x 25	-	-
	95	Feed alley	Biting midges	-	7	-	-

	96 - 99	Walkway	Culicine mosquitoes	-	4 x 1	-	-
	100 - 103	Feed alley	Culicine mosquitoes	-	4 x 1	-	-
	104 + 105	Outside	Culicine mosquitoes	-	2 x 1	-	-
	106	Aerial sweep net	Horseflies	-	1	-	-

**Month**	**Labeling on Eppendorf tubes**	**Trap location**	**Arthropod species**	**Fed**	**Uncertain**	**Unfed**	**Destroyed**
**July 2022**	1	Walkway	*Stomoxys*	-	1	-	-
	2	Outside	*Stomoxys*	-	1	-	-
	3	Feed alley	*Stomoxys*	-	1	-	-
	4 - 48	Aerial sweep net	*Stomoxys*	-	45 x 5	-	-
	49	Aerial sweep net	*Stomoxys*	-	2	-	-
	50 - 64	Aerial sweep net	*Stomoxys*	15 x 5	-	-	-
	65	Aerial sweep net	*Stomoxys*	1	-	-	-
	1	Aerial sweep net	Biting midges	-	1	-	-
	2	Feed alley	Biting midges	1 x 25	-	-	-
	3	Feed alley	Biting midges	4	-	-	-
	4 - 19	Feed alley	Biting midges	-	-	16 x 25	-
	20	Feed alley	Biting midges	-	-	5	-
	21 - 54	Feed alley	Biting midges	-	34 x 25	-	-
	55	Walkway	Biting midges	1 x 25	-	-	-
	56	Walkway	Biting midges	6	-	-	-
	57 - 66	Walkway	Biting midges	-	-	10 x 25	-

	67	Walkway	Biting midges	-	-	5	-
	68 - 103	Walkway	Biting midges	-	36 x 25	-	-
	104	Walkway	Biting midges	-	18	-	-
	105	Outside	Biting midges	1 x 25	-	-	-
	106	Outside	Biting midges	13	-	-	-
	107 - 118	Outside	Biting midges	-	-	12 x 25	-
	119	Outside	Biting midges	-	-	16	-
	120 - 148	Outside	Biting midges	-	29 x 25	-	-
	149	Outside	Biting midges	-	1 x 25	-	-
	149 **a**	Outside	Biting midges	-	13	-	-
	150 - 178	Outside	Culicine mosquitoes	-	29 x 1	-	-
	179	Outside	Anopheline mosquitoes	-	1	-	-
	180 - 188	Walkway	Culicine mosquitoes	-	9 x 1	-	-
	189 - 205	Feed alley	Culicine mosquitoes	-	17 x 1	-	-
	206 - 208	Feed alley	Anopheline mosquitoes	-	3 x 1	-	-
	209 + 210	Aerial sweep net	Horseflies	-	2 x 1	-	-
	211	Aerial sweep net	*Simulium*	-	1	-	-
**Month**	**Labeling on Eppendorf tubes**	**Trap location**	**Arthropod species**	**Fed**	**Uncertain**	**Unfed**	**Destroyed**
**August 2022**	1	Walkway	*Stomoxys*	-	2	-	-
	2	Feed alley	*Stomoxys*	-	3	-	-
	3	Feed alley	*Stomoxys*	1	-	-	-
	4	Outside	*Stomoxys*	-	1	-	-

	5 - 66	Aerial sweep net	*Stomoxys*	-	62 x 5	-	-
	67 - 82	Aerial sweep net	*Stomoxys*	16 x 5	-	-	-
	83	Aerial sweep net	*Stomoxys*	3	-	-	-
	84 - 85	Aerial sweep net	*Stomoxys*	-	2 x 5	-	-
	86	Aerial sweep net	*Stomoxys*	-	1	-	-
	1	Feed alley	Biting midges	1 x 25	-	-	-
	2	Feed alley	Biting midges	2	-	-	-
	3 + 4	Feed alley	Biting midges	-	-	2 x 25	-
	5	Feed alley	Biting midges	-	-	16	-
	6 - 51	Feed alley	Biting midges	-	46 x 25	-	-
	52	Feed alley	Biting midges	-	6	-	-
	53	Outside	Biting midges	11	-	-	-
	54 - 56	Outside	Biting midges	-	-	3 x 25	-
	57	Outside	Biting midges	-	-	21	-
	58 - 76	Outside	Biting midges	-	19 x 25	-	-
	77	Outside	Biting midges	-	22	-	-
	78	Walkway	Biting midges	21	-	-	-
	79 - 84	Walkway	Biting midges	-	-	6 x 25	-
	85	Walkway	Biting midges	-	-	10	-
	86 - 123	Walkway	Biting midges	-	38 x 25	-	-
	124	Walkway	Biting midges	-	15	-	-
	124 a	Aerial sweep net	Biting midges	2	-	-	-

	125 - 133	Walkway	Culicine mosquitoes	-	9 x 1	-	-
	134 - 139	Outside	Culicine mosquitoes	-	6 x 1	-	-
	140 - 149	Feed alley	Culicine mosquitoes	-	10 x 1	-	-
	150 - 157	Feed alley	*Simulium*	-	8 x 5	-	-
	158	Feed alley	*Simulium*	-	1	-	-
	159 - 166	Walkway	*Simulium*	-	8 x 5	-	-
	167	Walkway	*Simulium*	-	4	-	-
**Month**	**Labeling on Eppendorf tubes**	**Trap location**	**Arthropod species**	**Fed**	**Uncertain**	**Unfed**	**Destroyed**
**September 2022**	1 - 26	Aerial sweep net	*Stomoxys*	-	26 x 5	-	-
	27	Aerial sweep net	*Stomoxys*	-	1	-	-
	28 - 34	Aerial sweep net	*Stomoxys*	7 x 5	-	-	-
	35	Aerial sweep net	*Stomoxys*	1	-	-	-
	36	Feed alley	Biting midges	1	-	-	-
	37	Feed alley	Biting midges	-	-	7	-
	38	Feed alley	Biting midges	-	1 x 25	-	-
	39	Feed alley	Biting midges	-	8	-	-
	40	Walkway	Biting midges	3	-	-	-
	41	Walkway	Biting midges	-	-	9	-
	42	Walkway	Biting midges	-	1 x 25	-	-
	43	Outside	Biting midges	-	4	-	-
	44 + 45	Outside	Culicine mosquitoes	-	2 x 1	-	-
	46	Feed alley	*Simulium*	-	1	-	-
